# Understanding the patterns of superficial veins in cubital region: a cross-sectional study from Gulf Arab Populations

**DOI:** 10.12688/f1000research.185084.1

**Published:** 2026-07-04

**Authors:** Abdel Halim Salem, Razan Yusuf Alkhatib, Reem Yousef Almarshad, Turki Fahad Alshahrani, Munirah Kateb Alshammari, Wedad S. Al Muhaish, Wael Amin Nasr El-Din, Bhagath Kumar Potu

**Affiliations:** 1 Department of Anatomy, Arabian Gulf University College of Medicine and Health Sciences, Manama, Bahrain; 2 Department of Neurology, King Fahad Military Medical Complex, Dhahran, Dhahran, Eastern Province, Saudi Arabia; 3 Department of Neurosurgery, Imam Abdulrahman Bin Faisal University, Dammam, Dammam, Saudi Arabia; 4 Department of Pediatric Pulmonology, Children's Specialized Hospital, King Fahad Medical City, Riyadh, Saudi Arabia; 5 Department of Obstetrics and Gynecology, Security Forces Hospital Dammam, Dammam, Saudi Arabia; 6 Arabian Gulf University College of Medicine and Health Sciences, Manama, Bahrain; 7 Department of Anatomy, Arabian Gulf University College of Medicine and Health Sciences, Manama, Bahrain; 8 Department of Anatomy, Arabian Gulf University College of Medicine and Health Sciences, Manama, Bahrain

**Keywords:** superficial veins, cubital region, variations, venous patterns, Gulf Arab, population.

## Abstract

**Background:**

Superficial veins in the cubital region are important for medical practitioners to access the circulatory system for various clinical procedures. Despite their clinical importance, these veins are known to exhibit constant variations in their patterns from population-to- population. This prompted us to explore the various venous patterns in the cubital region of the Gulf Arab population.

**Methods:**

A cross-sectional study was conducted at the Arabian Gulf University, Bahrain, involving medical students from Saudi Arabia, Bahrain, and Kuwait. This study included a total number of volunteers (n = 166). A total of 332 venous patterns in the cubital region were recorded by applying a tourniquet, using a standard protocol.

**Results:**

The volunteers who participated in this study were the range of 17–27 years. Of the total participants, the majority were males (n = 139; 83.73%) with a lower number of females (n = 27; 16.27%) presenting 9 different types of venous patterns with type 6 venous pattern (29.2%) as the most common, followed by type 1 venous pattern (24.09%) in both males and females and in right-left sides. Type 5 was the least common variant observed in 0.3% of cases. Overall, no statistically significant differences were found between the variant venous patterns of the Bahraini-Saudi, Bahraini–Kuwaiti, and Saudi-Kuwaiti populations (P > 0.05).

**Conclusion:**

Our findings reveal that type 6 is the most common venous pattern, followed by type 1, in the cubital regions of both males and females of the studied populations. These observations are unique and inconsistent with the findings of earlier studies. Sound knowledge of these variant venous patterns is very useful when performing safe venipuncture and complex surgical interventions.

## Introduction

### Background

The upper limb veins are anatomically categorized into superficial and deep groups. The superficial veins are located subcutaneously within the superficial fascia of the arm, and the deep veins are those accompanying the major arteries in the form of venae comitantes. ^1^ Superficial veins are highly variable in their anatomy and clinically important as they can affect the success of venipuncture, transfusions, intravenous injections, cardiac catheterization, and the placement of vascular access in microsurgical procedures. ^2^ The superficial venous drainage from the upper limb occurs through two or three main superficial veins, which can differ significantly in their site of origin and course. ^2^ The primary superficial veins of the upper limb are the Cephalic and Basilic veins. These two veins are connected to each other by the median cubital vein in the cubital fossa. ^1^ Although this basic anatomical scheme of venous drainage in the area of cubital fossa is well described in classic anatomy textbooks, numerous studies investigating the venous variations over the past decades conveniently concluded that the venous patterns in this area are extremely variable and often pose challenges to the phlebologists. ^3^ Recognizing these venous variations is crucial for medical students, educators and healthcare professionals, as standard anatomy textbooks often oversimplify the complexities found in real clinical scenarios. ^2^

Recent years have seen a significant rise in interest in studying the variations in venous networks in the cubital region, which stems from numerous technologies evolving in the fields of endovascular surgery and transplantation medicine. Topographic studies, cadaveric dissections, radiologic evaluations, and angiographic procedures have shown a wide range of venous variants in the superficial veins of the cubital region, and the frequency of these variants is highly debatable from population-to-population with different methodologies used in evaluating them. ^3^ ^–^ ^5^ From an educational standpoint, we conducted this study to analyze the variant venous patterns in the cubital region of healthy volunteers from Saudi Arabia, Bahrain, and Kuwait populations.

## Methods

### Study participants

Convenience sampling for this cross-sectional study was conducted at Arabian Gulf University, Bahrain, involving medical students from Saudi Arabia, Bahrain, and Kuwait. This study included a total of 166 volunteers (139 being males and 27 females). Participants who had soft tissue injuries, fractures in the region of the cubital fossa, and those who were highly obese or whose superficial veins were not clearly palpable were excluded. Participation in this study was voluntary, and all the participants were informed about the study. Since this was an observational study and did not involve any major surgical or procedural interventions involved, verbal consent was obtained from all study participants before the procedure. Study has been performed in accordance with the principles stated in the Declaration of Helsinki.

### Procedure for examining the venous patterns

**Figure 1.**
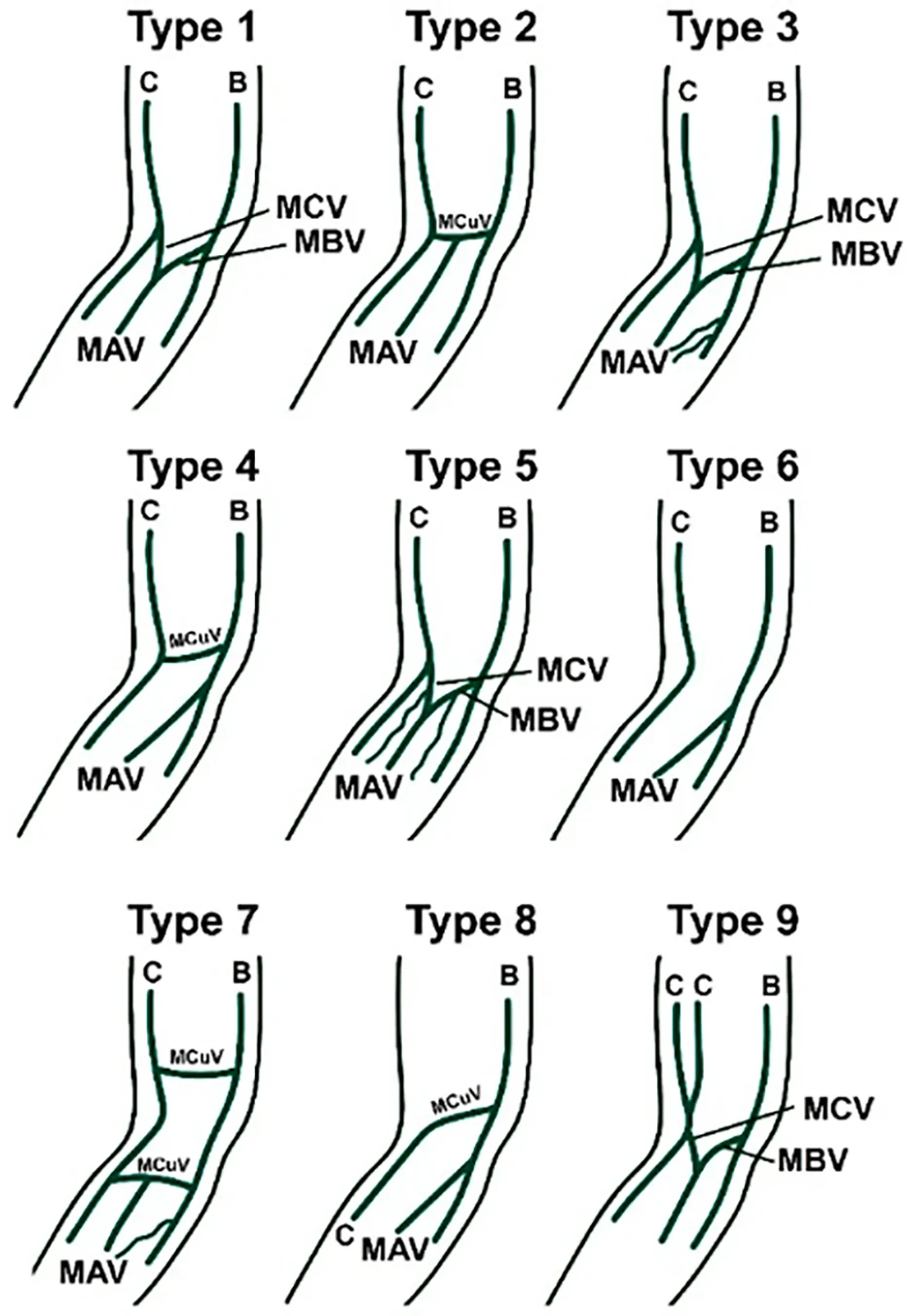
Showing 9 different types of venous patterns of the cubital region. C: Cephalic vein; B: Basilic vein; MCV: median cephalic vein; MBV: median basilic vein; MAV: median antebrachial vein; MCuV: median cubital vein.

The venous patterns of 139 males and 27 females were recorded following a standard protocol described by Vucinic et al., 2016. ^3^ In brief, a tourniquet was placed approximately 10 cm proximal to the elbow crease for about 3 to 5 minutes with repeated clenching and unclenching of their fists until the superficial veins were dilated and palpable for observation. Once the veins were dilated and palpable, the investigators collected the data and recorded the observed venous pattern following a standard classification as described by Vucinic et al., 2016. ^3^ In brief. This classification ( Figure 1) described nine types of venous patterns in the cubital region as follows:

**Type 1** presenting “M” shaped pattern, in which the median antebrachial vein continues with the two terminal branches of the median cephalic and median basilic vein, which connects the cephalic and the basilic vein, respectively.

**Type 2** presenting “N” shaped pattern, in which the median antebrachial vein ends into median cubital vein.

**Type 3** presenting more or less similar to “M shaped pattern”.

**Type 4** presenting a modified “N shaped pattern,” in which, median antebrachial vein ends in the basilic vein.

**Type 5** presenting modified “M shaped pattern” **,** in which, one or two veins end either in median cephalic or median basilic vein.

**Type 6** presents the cephalic and basilic veins with no communication between them, and the median antebrachial vein opens into either the basilic or cephalic vein.

**Type 7** presents two median cubital veins above and below the crease of the elbow, with the median antebrachial vein opening into the medial cubital vein or basilic vein.

**Type 8** presents with a cephalic vein running supermedially as the median cubital vein with no proximal cephalic vein.

**Type 9** presenting “M shaped pattern” with a doubled brachial cephalic vein.

### Statistical analysis

The data collected were analyzed using the Statistical Package for Social Sciences (SPSS) version 30 (Chicago, IL, USA). Descriptive statistics were used to determine the frequencies and percentages of the different types of venous patterns. Associations between the types of venous pattern, sex, and limb laterality were assessed using the Chi-Square and Fisher’s exact tests. Statistical significance was set at p < 0.05.

## Results

**Figure 2.**
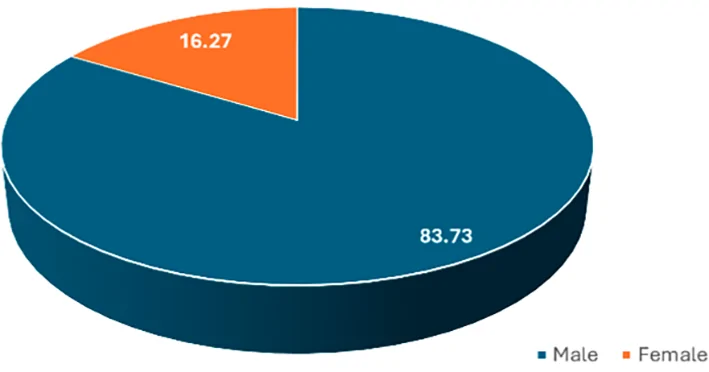
Pie Chart showing the percentage of male and female participants of the study.

**Figure 3.**
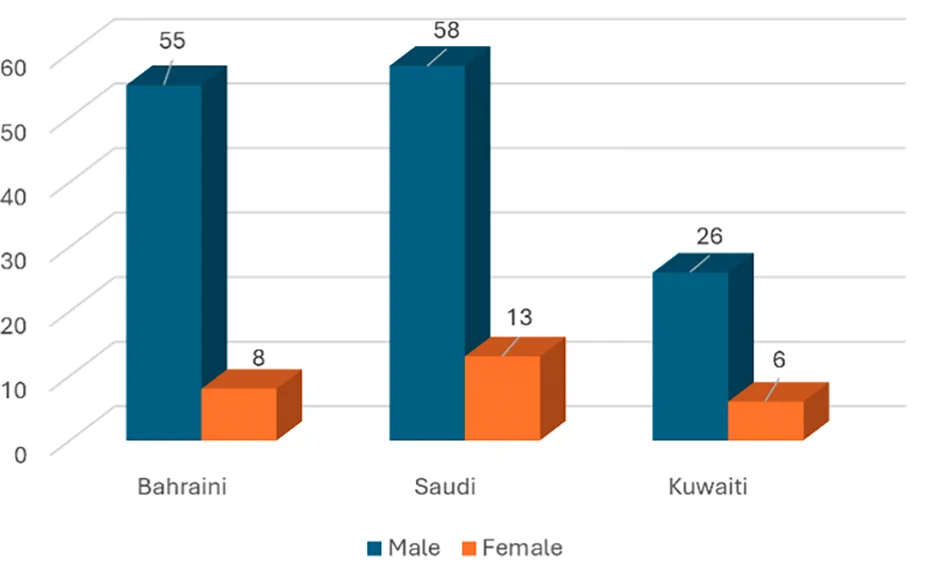
Number of male and female participants from different Gulf Arab populations.

The age of the volunteers who participated in this study was in the range of 17–27 years. The mean age of the participants was 20 ± 2.17. Of the total participants, the majority were male (83.73%), with a lower number of females (16.27%) ( Figure 2). Of these 139 males, 55 were from Bahrain, 58 were from Saudi Arabia, and 26 were from Kuwait. Of the 27 females, 8 were from Bahrain, 13 from Saudi Arabia, and 6 from Kuwait ( Figure 3, Tables 1 and 2).

**Table 1.** Venous patterns of the superficial veins in cubital region with regards to gender and laterality.

Pattern type	Males				Females			
	Rt CR	Lt CR	Total	%	Rt CR	Lt CR	Total	%
Type 1	28	34	62	22.30	12	6	18	33.33
Type 2	9	7	16	5.76	0	0	0	0
Type 3	22	23	45	16.19	1	2	3	5.56
Type 4	13	12	25	8.99	4	2	6	11.11
Type 5	16	12	28	10.07	0	0	0	0
Type 6	40	37	77	27.70	8	12	20	37.04
Type 7	0	1	1	0.36	0	0	0	0
Type 8	3	3	6	2.16	2	2	4	7.41
Type 9	8	10	18	6.47	0	3	3	5.56
Total	139	139	278	100	27	27	54	100

*Note*: Rt CR: right cubital region; Lt CR: left cubital region

**Table 2.** Number of limbs, gender and laterality of various venous patterns studied in different Gulf Arab populations.

		Males				Females			
		Rt CR	Lt CR	Total	%	Rt CR	Lt CR	Total	%
**Bahrain**	Type 1	12	13	25	22.73	5	3	8	50.00
	Type 2	1	2	3	2.73	0	0	0	0
	Type 3	7	6	13	11.82	0	0	0	0
	Type 4	6	6	12	10.91	0	0	0	0
	Type 5	8	4	12	10.91	0	0	0	0
	Type 6	17	18	35	31.82	2	3	5	31.25
	Type 7	0	1	1	0.91	0	0	0	0
	Type 8	0	1	1	0.91	1	2	3	18.75
	Type 9	4	4	8	7.27	0	0	0	0.00
	Total	55	55	110	100.00	8	8	16	100.00
**Saudi Arabia**		**Males**				**Females**			
		Rt CR	Lt CR	Total	%	Rt CR	Lt CR	Total	%
	Type 1	8	12	20	17.24	6	3	9	34.62
	Type 2	6	3	9	7.76	0	0	0	0.00
	Type 3	10	12	22	18.97	0	1	1	3.85
	Type 4	4	6	10	8.62	3	2	5	19.23
	Type 5	6	6	12	10.34	0	0	0	0.00
	Type 6	18	13	31	26.72	4	6	10	38.46
	Type 7	0	0	0	0.00	0	0	0	0.00
	Type 8	3	2	5	4.31	0	0	0	0.00
	Type 9	3	4	7	6.03	0	1	1	3.85
	Total	58	58	116	100.00	13	13	26	100.00
**Kuwait**		**Males**				**Females**			
		Rt CR	Lt CR	Total	%	Rt CR	Lt CR	Total	%
	Type 1	8	9	17	32.69	1	0	1	8.33
	Type 2	2	2	4	7.69	0	0	0	0
	Type 3	5	5	10	19.23	1	1	2	16.67
	Type 4	3	0	3	5.77	1	0	1	8.33
	Type 5	2	2	4	7.69	0	0	0	0
	Type 6	5	6	11	21.15	2	3	5	41.67
	Type 7	0	0	0	0.00	0	0	0	0
	Type 8	0	0	0	0.00	1	0	1	8.33
	Type 9	1	2	3	5.77	0	2	2	16.67
	Total	26	26	52	100	6	6	12	100

*Note*: Rt CR: right cubital region; Lt CR: left cubital region

**Type 1** was identified in 40 right (28 males +12 females) and 40 cubital regions (34 males and 6 females). Eighty cubital regions exhibited this venous pattern. No significant differences were noted between the sexes (p = 0.109) or laterality (p = 1).

**Type 2** was identified in nine right and seven left cubital regions of males with 0% females. Sixteen cubital regions exhibited this pattern. Statistically significant differences were noted between the sexes (p = 0). However, no statistically significant differences were noted in laterality (p = 0.608).

**Type 3** was identified in 23 right cubital regions (22 males and 1 female) and 25 left cubital regions (23 males and 2 females). A total of 48 cubital regions exhibited this venous pattern. Statistically significant differences were noted between sexes (p = 0.005). However, no statistically significant differences were noted in laterality (p = 0.755).

**Type 4** was identified in 17 right cubital regions (13 males and 4 females) and 14 left cubital regions (12 males and 2 females). A total of 31 cubital regions exhibited this venous pattern. No significant differences were noted between the sexes (p = 0.646) or laterality (p = 0.571).

**Type 5** was identified in 16 right (16 males +0 females) and 12 left cubital regions (12 males and 0 females). A total of 28 cubital regions exhibited this venous pattern. Statistically significant differences were noted between the sexes (p = 0). However, no statistically significant differences were noted in laterality (p = 0.429).

**Type 6** was identified in 48 right cubital regions (40 males and 8 females) and 49 left cubital regions (37 males and 12 females). In total, 97 cubital regions exhibited this venous pattern. No significant differences were noted between the sexes (p = 0.188) or laterality (p = 0.904).

**Type 7** was identified in only 1 male patient. Statistically significant differences were noted between the sexes (p = 0). However, no statistically significant differences were noted in laterality (p = 0.316).

**Type 8** was identified in five right cubital regions (three males and two females) and five left cubital regions (three males and two females). Ten cubital regions exhibited this pattern. No significant differences were noted between the sexes (p = 0.153) or laterality (p = 1).

**Type 9** was identified in 8 right cubital regions (8 males and 0 females) and 13 left cubital regions (10 males and 3 females). A total of 21 cubital regions exhibited this venous pattern. No significant differences were noted between the sexes (p = 0.79) or laterality (p = 0.259).

Overall, no statistically significant differences were found among the Bahraini-Saudi, Bahraini – Kuwaiti, and Saudi-Kuwaiti populations (p > 0.05). Our findings on the various venous patterns have been compared with other studies available in the literature ( Table 3).

**Table 3.** Venous patterns reported from different populations following a standard classification described by Vučinić et al. ^3^

Study & Year	Population	Sample size (cubital regions)	Type 1	Type 2	Type 3	Type 4	Type 5	Type 6	Type 7	Type 8	Type 9
Del Sol et al. ^4^	Chilian	40	12 (30%)	12 (30%)	10 (25%)	4 (10%)	-	-	-	2 (5%)	-
Dharap and Shaharuddin ^5^	Malaysian	532	86 (16.2%)	362 (68.%)	44 (8.3%)	12 (2.2%)	1 (0.6%)	26 (4.9%)	-	-	-
Jasiński and Poradnik ^6^	Polish	80	26 (32.5%)	35 (43.7%)	-	13 (16.2%)	-	-	-	6 (7.5%)	-
Vasudha ^7^	Indian	50	44 (88%)	2 (4%)	2 (4%)	2 (4%)	-	-	-	-	-
Mikuni et al ^8^	Japanese	128	1 (0.78%)	104 (82%)	9 (7%)	-	-	-	-	14 (11%)	-
Ukoha et al ^9^	Nigerian	270	75 (27.8%)	76 (28.2%)	11 (4.1%)	14 (5.2%)	-	-	14 (5.2%)	80 (29.6%)	-
Bekel et al. ^10^	Ethiopian	800	468 (58.5%)	149 (18.6%)	112 (14%)	71 (8.9%)	-	-	-	-	-
Ghasem et al. ^11^	Iranian	620	103 (19.43%)	182 (34.3%)	141 (26.6%)	83 (15.6%)	8 (1.5%)	11 (2.07%)	2 (0.37%)	-	-
Melaku et al. ^12^	Ethiopian	802	206 (25.7%)	441 (55%)	81 (10.1%)	53 (6.6%)	21 (2.6%)	-	-	-	-
Hiware et al. ^13^	Eastern Province, Saudi	247	80 (32.4%)	129 (52.2%)	21 (8.5%)	16 (6.5%)	1 (0.4%)	-	-	-	-
Elghazaly ^14^	Sudanese	290	81 (27.9%)	67 (23.1%)	66 (22.8%)	66 (22.8%)	10 (3.4%)	-	-	-	-
Jahan et al. ^15^	Sri Lankan	30	10 (33.3%)	4 (13.34%)	9 (30%)	3 (10%)	-	1 (3.33%)	-	-	-
Our study As per standard classification described by Vucinic et al. ^3^	Saudi, Bahraini & Kuwaiti	332	80 (24.09%)	16 (4.8%)	48 (14.4%)	31 (9.3%)	28 (8.4%)	97 (29.2%)	1 (0.3%)	10 (3%)	21 (6.3%)
Vučinić et al. ^3^	Serbs	338	115 (34.02%)	97 (28.6%)	42 (12.4%)	28 (8.2%)	20 (5.9%)	17 (5.02%)	10 (2.9%)	6 (1.7%)	3 (0.8%)

## Discussion

The structural and visual differences observed in the superficial venous patterns of the cubital region are driven by many factors, including biological and environmental factors such as embryological development, genetic predisposition, body composition, and demographic differences. ^7^ During embryogenesis, veins of the upper limb originate as a capillary network and undergo a series of changes. During this remodeling process, many veins enlarge to form the main veins, while others regress. Any deviations during this normal process of regression and enlargement would present variant types of superficial veins that are largely determined by genetics, which dictate the specific branching pattern, size, and anastomoses of vascular networks. ^7^

The current study conducted on superficial veins of the cubital region in the Gulf Arab population reported that the type 6 venous pattern (29.2%) was the most common, followed by the type 1 venous pattern (24.09%) in both males-females and on the right-left side. The occurrence of all other venous pattern types was significantly lower than that of these two types. Of all the types reported, type 7 was the rarest, reported in only one cubital region in the male and on the left side.

Our literature search concluded that there was only one study conducted in a Gulf Arab population from the eastern province of Saudi Arabia, ^13^ which reported only four types of venous patterns following the classification described by Lee et al. ^16^ This classification is entirely different from criterion ^3^ we followed in our study, which makes our study of its first kind in reporting the detailed superficial venous patterns of the cubital region. According to their study ^3^ and classification, type 2 was the most common (52.2%), followed by type 1 (32.4%). There are three studies available in the literature that report superficial venous patterns of the cubital region in Pan-Arab populations. A study from a Jordanian population following the classification proposed by Del Sol et al. ^4^ reported that the type 2 pattern was the most common pattern followed by type 1. ^17^ Another study from the Iraqi population reported an M-shaped pattern (type 1) as the most common pattern followed by type 2 as per the classification we followed in our study. ^18^ A study from the Egyptian population revealed an N-shaped pattern (type 2) as the most common venous pattern. ^19^ These findings are contrary to our results, and this could be due to the variation in the pool of the populations studied. ^7^

A study from the Malaysian population ^5^ reported six venous patterns in 532 cubital regions. The most common type reported in their study was type 2 with an N-shaped arrangement (68%) in 362 cubital regions, followed by an M-shaped arrangement in 86 cubital regions (16.2%). Studies from Polish, Japanese, Nigerian, and Iranian populations also followed the same trend, reporting more N-shaped venous patterns. ^6^ ^,^ ^8^ ^,^ ^9^ ^,^ ^11^ However, studies from Serbian, Indian, Sudanese, and Sri Lankan populations presented more M-shaped (type 1) venous patterns than the N-shaped arrangement. ^3^ ^,^ ^7^ ^,^ ^14^ ^,^ ^15^ These venous patterns reported from different populations follow a standard anatomy textbook description of type 2 being the most common, wherein the cephalic vein gives off the median cubital vein, which passes upward and medially to join the basilic vein. However, our study does not support this view, as type 6 is the most common. A study from the Ethiopian population ^10^ conducted on 800 cubital regions presented a different scenario of type 1 being the commonest than the type 2. Contrary to this, another study from the Ethiopian population with more or less the same sample revealed type 2 being the most common one than type 1. ^12^ These variations observed from the same populations or comparing them with different populations are highly debatable in the clinical context, as these venous patterns depend on the hydration status, gender, genetic predisposition, age, and thickness of the subcutaneous fatty layer of the individuals. ^3^ A thorough understanding of all the reported venous patterns and their predominance are crucial for healthcare workers, medical lab technicians, and phlebologists to improve their venipuncture practice and to avoid any procedural complications. ^2^ It is essential to spot and accurately identify the basilic vein and median cubital vein, particularly during the procedures of dialysis and venipuncture ^20^ and bearing the fundamental knowledge of variant venous patterns reported in our study and other comprehensive studies ^3^ ^,^ ^21^ would avoid any pressing challenges associated with these clinical procedures.

This study has several limitations. First, we did not obtain an equal number of subjects of either sex. Second, we did not correlate venous patterns with anthropometric variables such as height, weight, or body mass index (BMI). In the future, it may be worth examining these parameters to gain a more comprehensive understanding of the venous anatomy and to synthesize contemporary data about fundamental knowledge of the superficial veins of the cubital region, as these veins hold the highest value in diagnostic, therapeutic, and surgical procedures.

In conclusion, our study observed that the type 6 being the commonest venous pattern followed by the type 1 in the cubital regions of both males and females. These observations are unique and inconsistent with the findings of earlier studies.

## Ethical approval

This study was approved by the Research & Ethics Committee (REC) of the College of Medicine & Health Sciences (CMHS), Arabian Gulf University (AGU) (Approval no: E41-PI-06-26).

## Data Availability

All the results and data are available in this manuscript, and extended data are available at https://doi.org/10.6084/m9.figshare.32770011. ^22^ Data are available under the terms of the Creative Commons Zero “No rights reserved data waiver (CC0 1.0 Public domain dedication).
