# Supplementary material for: Understanding the patterns of superficial veins in cubital region: a cross-sectional study from Gulf Arab Populations
Source: F1000Res. 2026 Jul 4;15:1068. [Version 1] doi: 10.12688/f1000research.185084.1 (PMC13550921; doi:10.12688/f1000research.185084.1)
Supplement: Peer review history

# Reviewer report: Reviewer response for version 1

**DOI:** 10.5256/f1000research.204299.r499380

**Published:** 2026-07-11

**Authors:** Abdelmonem Awad Hegazy

**Affiliations:** 1 Zagazig University, Zagazig City, Egypt

Abstract:

 Please specify the details of the mentioned type; simply referring to "Type 6" is not sufficient.

 Introduction

 There is no need to repeat the reference number in the same sequence within the text; for example, reference number 2 can be cited once at the end of the relevant text.

 Methods

 The inclusion criteria have not been sufficiently clarified; please add the details.

 Please add the website link for this "Declaration of Helsinki.".

 This phrase (Type 3 presenting more or less similar to “M shaped pattern”.) is ambiguous. Please rephrase it clearly.

 The source of Figure 1 must be cited in the Acknowledgements section. 

 Results

 This phrase "participants from different Gulf Arab populations." can be modified to "participants from some Gulf Arab populations.".

 Illustrative photographs showing the different patterns of vein distribution among the participants must be included, without revealing their names or details.

 This sentence (Our findings on the various venous patterns have been compared with other studies available in the literature) does not express a result but rather describes the methodology; therefore, you can move it to the methodology section and state the key results instead.

 Discussion

 If this paragraph (The current study conducted on superficial veins of the cubital region in the Gulf Arab population reported that the type 6 venous pattern (29.2%) was the most common, followed by the type 1 venous pattern (24.09%) in both males-females and on the right-left side. The occurrence of all other venous pattern types was significantly lower than that of these two types. Of all the types reported, type 7 was the rarest, reported in only one cubital region in the male and on the left side.) does not include a discussion of other studies, you may delete it, as it constitutes a repetition.

 You can add the following:

 The present study investigated the venous pattern of the cubital fossa to evaluate its suitability for upper-limb venous access. Other researchers have proposed using the area posterior to the anterior border of the distal radius as an anatomical landmark to locate the site for venous access via the cephalic vein.

 Ref 1: Hegazy EA, Khalil M. An anatomical study of the cephalic vein in the forearm. New Egypt J Med. 2006;35:77-80

 Study limitations may also include the small sample size of participants; furthermore, the current study did not employ imaging techniques to visualize venous anatomy.

Is the work clearly and accurately presented and does it cite the current literature?

Yes

If applicable, is the statistical analysis and its interpretation appropriate?

Yes

Are all the source data underlying the results available to ensure full reproducibility?

Yes

Is the study design appropriate and is the work technically sound?

Yes

Are the conclusions drawn adequately supported by the results?

Yes

Are sufficient details of methods and analysis provided to allow replication by others?

Yes

Reviewer Expertise:

Human Anatomy, Embryology

I confirm that I have read this submission and believe that I have an appropriate level of expertise to confirm that it is of an acceptable scientific standard.

# Reviewer report: Reviewer response for version 1

**DOI:** 10.5256/f1000research.204299.r499383

**Published:** 2026-07-10

**Authors:** Dr. Vasantha kumar Pakirisamy

**Affiliations:** 1 Department of Anatomy, St. Matthew's University School of Medicine, West Bay, Cayman Islands

1. **Consistency in terminology:**
  The terminology should be standardized throughout the manuscript. Please use either **“venous pattern”** or **“venous arrangement”** consistently to avoid ambiguity and improve clarity.
2. **Refinement of the conclusion statement:**
  The statement *“These observations are unique and inconsistent with earlier studies”* may be interpreted as overly strong. A more scientific and balanced interpretation would be:

*"In conclusion, the present study identified type 6 as the predominant superficial venous pattern of the cubital region in the Gulf Arab population, followed by type 1. This finding differs from the patterns commonly reported in previous anatomical studies, where type  (mention the pattern) and type  (mention the pattern) have frequently been described as the predominant venous pattern in several populations."*

*3.* **Illustration of type 6 venous pattern:**

 If available, inclusion of a representative image or schematic illustration of the **type 6 superficial venous pattern** would improve the clarity and clinical relevance of the manuscript. A visual representation would help readers better understand this relatively uncommon venous pattern and appreciate its anatomical characteristics.

Is the work clearly and accurately presented and does it cite the current literature?

Yes

If applicable, is the statistical analysis and its interpretation appropriate?

Yes

Are all the source data underlying the results available to ensure full reproducibility?

Yes

Is the study design appropriate and is the work technically sound?

Yes

Are the conclusions drawn adequately supported by the results?

Yes

Are sufficient details of methods and analysis provided to allow replication by others?

Yes

Reviewer Expertise:

Anatomy, radiology, anthropometry,

I confirm that I have read this submission and believe that I have an appropriate level of expertise to confirm that it is of an acceptable scientific standard.
